# The Impact of Dental Prostheses in Patients With Cardiovascular Disease: A Scoping Review With Exploratory Analysis

**DOI:** 10.1016/j.identj.2026.109691

**Published:** 2026-06-19

**Authors:** Monika Werdiningsih, En-Chih Weng, Yoko Hasegawa, Ma. Therese Sta. Maria, Kazuhiro Hori, Yasuyuki Nagasawa, Ken Shinmura

**Affiliations:** a Division of Comprehensive Prosthodontics, Faculty of Dentistry & Graduate School of Medical and Dental Sciences, Niigata University, Niigata, Japan; b Department of Dental Public Health and Preventive Dentistry, Faculty of Dentistry, Universitas Indonesia, Jakarta, Indonesia; c Department of Dentistry and Oral Surgery, Hyogo College of Medicine, Nishinomiya, Hyogo, Japan; d Department of Prosthodontics, Faculty of Dentistry, Manila Central University, Caloocan, Philippines; e Department of General Internal Medicine, School of Medicine, Hyogo Medical University, Nishinomiya, Hyogo, Japan

**Keywords:** Dental prostheses, Cardiovascular disease, Hypertension, Frailty, Physical function

## Abstract

Dental prostheses not only restore oral function but may also impact systemic health outcomes. This scoping review sought to synthesize evidence on the association between dental prostheses, hypertension, and physical function, including frailty, in adults with established cardiovascular disease (CVD) or elevated cardiovascular risk. This review was conducted following the PRISMA-ScR guidelines and Joanna Briggs Institute methodology. Literature searches were performed in PubMed and Google Scholar up to November 10, 2024. Eligible studies were observational investigations involving adults with established CVD or elevated cardiovascular risk who used dental prostheses. An exploratory quantitative approach was used to summarize the direction and patterns of association. Seven studies met the inclusion criteria: 5 assessed hypertension and 2 examined physical function. Exploratory pooled analysis based on unadjusted data indicated that individuals with dental prostheses had a lower odds of hypertension (odds ratio = 0.69; 95% confidence interval, 0.52-0.91), although substantial heterogeneity (I² = 95%) and the use of unadjusted estimates imply a high risk of residual confounding. Limited evidence suggests that dental prosthesis use may help preserve mobility and mitigate frailty in patients with CVD; however, differences in frailty definitions and limited adjustment for confounding factors reduce comparability and indicate that these findings should be interpreted as exploratory and hypothesis-generating. Current evidence suggests that dental prosthesis use may offer benefits beyond oral rehabilitation, potentially linked to better hypertension-related outcomes and physical function in adults with CVD or elevated cardiovascular risk, but certainty is limited by the small number of studies and methodological heterogeneity. These exploratory, hypothesis-generating findings highlight the need for well-designed longitudinal studies to clarify potential associations and underlying mechanisms. Beyond restoring oral function, prosthodontic rehabilitation may have broader systemic implications, contributing to better cardiovascular-related outcomes in aging populations.

## Introduction

Cardiovascular disease (CVD) remains the foremost cause of death worldwide despite decades of progress in prevention and treatment.^1^ Multiple interacting risk factors, such as hypertension, malnutrition, physical inactivity, dyslipidemia, hyperglycemia, obesity, smoking, kidney dysfunction, and genetic predisposition, shape its onset and progression.^2^ Cardiovascular risk profiles vary by region and ethnicity. High blood pressure predominates in Asia, whereas the dual burden of obesity and high blood pressure is prominent in the United States.^3^^,^^4^

Among older adults, cardiovascular risk factors follow a distinct trajectory compared with those in younger populations.^5^^,^^6^ Age-related physiological changes, such as vascular aging and inflammation, together with lifelong lifestyle exposure, increase the susceptibility to hypertension and other CVD-related conditions.^7^, ^8^, ^9^ In this context, deterioration of oral health, particularly tooth loss and reduced masticatory function, can accelerate this trajectory by promoting malnutrition and elevating blood pressure. These changes may subsequently contribute to functional decline and frailty, which are recognized outcomes among patients with established CVD, rather than primary risk factors.^10^, ^11^, ^12^ Although hypertension is conventionally regarded as a major risk factor for cardiovascular disease, its role in older adults and in patients with established CVD differs from that of primary prevention.^13^ In these populations, blood pressure levels are routinely monitored and therapeutically modified as part of clinical management and therefore represent a modifiable outcome and an indicator of disease control rather than a static risk factor. Similarly, frailty in patients with CVD is increasingly recognized not as a predisposing risk factor but as a clinical outcome that reflects functional decline and disease progression.^14^ From this perspective, both blood pressure control and physical function can be regarded as clinically meaningful outcomes in aging populations with established cardiovascular disease (CVD) or elevated cardiovascular risk. Previous studies have emphasized that blood pressure control is closely linked to prognosis, functional status, and quality of life in these groups.^15^, ^16^, ^17^, ^18^

Dental prostheses serve not only as restorative interventions for oral function, but also as potential modulators of systemic health, linking oral physiology to cardiovascular and functional outcomes.^19^ By restoring mastication and supporting adequate dietary intake, dental prostheses may help regulate blood pressure, preserve muscle strength, and maintain mobility. Conversely, complications, such as denture stomatitis or shifts in the oral microbiome, may trigger systemic inflammation, potentially contributing to adverse cardiovascular outcomes.^19^^,^^20^

Although the connections between oral health and CVD outcomes are increasingly acknowledged, the specific contribution of dental prostheses to hypertension prevention and maintenance of physical function, particularly among patients with existing CVD, remains poorly defined. Most studies have focused on tooth loss, periodontal disease, or nutritional outcomes without investigating whether prosthodontic rehabilitation affects hypertension or physical function in patients with CVD.^21^^,^^22^

Accordingly, this scoping review aimed to synthesize current evidence on the association between dental prostheses and hypertension and physical function (including frailty as a clinical outcome) among individuals with or at risk for CVD. By repositioning frailty as an outcome rather than a cardiovascular risk factor, this study sought to clarify the potential link between prosthodontic rehabilitation, blood pressure regulation, and functional preservation in aging populations. An improved understanding of these relationships may help inform the integration of prosthodontic care into cardiovascular disease management and prevention.

## Materials and methods

### Protocol

This scoping review was designed and conducted in accordance with the Joanna Briggs Institute (JBI) methodological framework for scoping reviews. Reporting followed the Preferred Reporting Items for Systematic Reviews and Meta-Analyses Extension for Scoping Reviews (PRISMA-ScR) guidelines.^23^ A protocol was developed and registered in the Open Science Framework (OSF; registration ID: EAM3V) before data collection to enhance transparency and reproducibility.

### Research focus and questions

This review aimed to explore the impact of dental prostheses on hypertension and physical function as health outcomes among adults with or at risk for CVD. Frailty was not treated as a cardiovascular risk factor but rather as a clinical outcome reflecting physical decline in patients with CVD. Because the original search strategy was restricted to the term “physical function,” this review does not constitute a scoping review of frailty per se; however, 2 of the included studies incidentally reported frailty outcomes, which were integrated as part of the physical function domain.

Research questions were framed using the JBI-recommended Participant, Concept, and Context (PCC) approach, and additionally structured with the Participant, Intervention, Comparison, and Outcome (PICO) framework to enhance clinical interpretability.

The population consisted of adults with diagnosed or undiagnosed CVD, including those who developed CVD either before or after receiving dental prosthesis treatment. Intervention was defined as the use of dental prostheses to replace missing teeth and restore oral function. Dental prostheses are classified into 3 categories: (1) removable prostheses, such as partial or complete dentures that the patient can remove; (2) fixed prostheses, including crowns and bridges that are permanently attached to natural teeth or dental implants; and (3) implant-supported prostheses, which may be either fixed or removable and anchored by dental implants.

When studies did not specify the prosthesis type or retention mechanism, this was noted during data extraction. The comparison group included adults who did not wear any form of dental prosthesis.

The primary outcomes of this review were hypertension and physical function, both of which were considered clinical endpoints rather than cardiovascular risk factors. Physical function was broadly defined to encompass measures of mobility, performance, and frailty. Table 1 summarizes the 2 research questions (RQs) and their associated outcomes.

**Table 1:** Research questions and outcomes.

	Research question	Outcome
RQ1	Does treatment with dental prostheses influence blood pressure regulation?	Hypertension prevalence
RQ2	Does dental prostheses use improve or maintain physical function in adults with or at risk for CVD?	Mobility, physical performance, frailty

### Search strategies and literature selection criteria

Electronic searches were conducted in PubMed and Google Scholar on November 10, 2024, to identify all relevant publications. Google Scholar was included to ensure broad coverage of gray literature and conference outputs that might not appear in traditional databases. Search terms combined Medical Subject Headings (MeSH), title/abstract keywords, AND, free-text terms using Boolean operators (AND, OR). The full search strategy for each research question is presented in Table 2. As the original search strategy for RQ2 was restricted to the term “physical function,” the keywords “frailty” or “frail” were not included. Nevertheless, 2 studies incidentally reported frailty as an outcome and were, therefore, incorporated into the synthesis under the domain of physical function.

**Table 2:** Search formula for each research question.

**RQ1. Does treatment with dental prostheses influence blood pressure regulation?**	
	**Pubmed Search Strategy:**
#1	"cardiovascular diseases"[MeSH Terms] OR "heart diseases"[MeSH Terms] OR "stroke"[MeSH Terms] OR "cerebrovascular disorders"[MeSH Terms] OR "hypertension"[MeSH Terms]
#2	"heart disease"[Title/Abstract] OR "cerebrovascular disease"[Title/Abstract] OR "cerebrovascular accident"[Title/Abstract] OR "coronary heart disease"[Title/Abstract]
#3	(#1) OR (#2)
#4	"prosthodontics"[MeSH Terms] OR "dental prosthesis"[MeSH Terms] OR "dentures"[MeSH Terms] OR "denture, partial"[MeSH Terms] OR "denture, complete"[MeSH Terms] OR "denture, partial, removable"[MeSH Terms] OR "denture, partial, fixed"[MeSH Terms] OR "denture, overlay"[MeSH Terms] OR "dental prosthesis, implant supported"[MeSH Terms]
#5	"blood pressure"[MeSH Terms] OR "hypertension"[Title/Abstract] OR "systolic"[Title/Abstract] OR "diastolic"[Title/Abstract]
#6	(#3) AND (#4) AND (#5)

	**Google Scholar Search Strategy**:
#1	"Dental prosthesis"
#2	"cardiovascular disease"
#3	"blood pressure" OR "hypertension" OR "systolic" OR "diastolic"
#4	(#1) AND (#2) AND (#3)

**RQ2. Does dental prostheses use improve or maintain physical function in adults with or at risk for CVD?**	
	**Pubmed Search Strategy:**
#1	"cardiovascular diseases"[MeSH Terms] OR "heart diseases"[MeSH Terms] OR "stroke"[MeSH Terms] OR "cerebrovascular disorders"[MeSH Terms] OR "hypertension"[MeSH Terms]
#2	"heart disease"[Title/Abstract] OR "cerebrovascular disease"[Title/Abstract] OR "cerebrovascular accident"[Title/Abstract] OR "coronary heart disease"[Title/Abstract]
#3	(#1) OR (#2)
#4	"prosthodontics"[MeSH Terms] OR "dental prosthesis"[MeSH Terms] OR "dentures"[MeSH Terms] OR "denture, partial"[MeSH Terms] OR "denture, complete"[MeSH Terms] OR "denture, partial, removable"[MeSH Terms] OR "denture, partial, fixed"[MeSH Terms] OR "denture, overlay"[MeSH Terms] OR "dental prosthesis, implant supported"[MeSH Terms]
#5	“physical functional performance”[MeSH Terms] OR "mobility limitation"[MeSH Terms] OR "motor function"[Title/Abstract] OR "physical function"[Title/Abstract] OR "physical activity"[Title/Abstract] OR "functional performance"[Title/Abstract]
#6	(#3) AND (#4) AND (#5)

	**Google Scholar Search Strategy:**
#1	"Dental prosthesis"
#2	"cardiovascular disease"
#3	“physical function” OR "mobility" OR "physical activity" OR "motor function"
#4	(#1) AND (#2) AND (#3)

Notes: Frailty-related terms (“frailty” OR “frail”) were intentionally not included in the original search strategy for RQ2. This review focused on physical function as the predefined outcome domain.

Nevertheless, two of the included studies incidentally reported frailty outcomes, which were integrated into the synthesis as part of physical function to ensure completeness and transparency.

Searches were limited to English-language publications with full-text availability.

No date restrictions were applied prior to November 10, 2024.

The search process adhered to the Joanna Briggs Institute (JBI) recommendations and PRISMA-ScR reporting guidelines.

### Eligibility criteria

Studies were eligible if they met the following conditions: (1) were conducted in human populations, limited to adults with CVD or those at elevated cardiovascular risk; (2) investigated the role of dental prostheses, such as removable dentures (partial or complete), fixed prostheses, or implant-supported prostheses, in relation to either hypertension or physical function; (3) were published in English with both an abstract and full text available; and (4) employed any empirical design, including systematic reviews, meta-analyses, randomized controlled trials (RCTs), clinical studies, prospective or retrospective cohort studies, case-control studies, case reports, or conference abstracts.^23^

### Screening procedures

Two reviewers (“M.W.” and either “E.W.” or “M.M.”) independently evaluated titles and abstracts, followed by full-text screening according to the inclusion criteria. Discrepancies were resolved through discussion, and any remaining disagreements were adjudicated by a third reviewer (“Y.H.”).

### Data extraction and quality assessment

Data from the included studies were systematically extracted into standardized tables, capturing study characteristics including author names, title, year of publication, journal name, study objectives, study design, level of evidence, number of participants, follow-up period, and outcomes.

Methodological quality was assessed using the MINDS Manual for Guideline Development (2020) for Systematic Review, adapted from the GRADE (Grading of Recommendations, Assessment, Development, and Evaluation) approach.^24^ The following 5 domains were evaluated: Risk of bias, inconsistency, indirectness, imprecision, and publication bias. For observational studies, potential sources of bias include selection, information, detection, and attrition. Bias ratings were assigned on a 3-point scale: high risk (-2), unclear (-1), or low risk (0).

The overall risk of bias was summarized based on the predefined domains. A majority of -2 was interpreted as a very serious risk, a mixed judgment indicated a serious risk, and a majority of 0 indicated no apparent risk. These domain-specific judgments were then incorporated into the GRADE framework to determine the certainty of the evidence for each outcome. Certainty was rated high, moderate, low, or very low. Outcomes were rated as very low if one or more domains presented very serious concerns, and as low when concerns were serious but not very serious. These processes enabled a transparent appraisal of confidence in the evidence base.^25^

Statistical analyses were conducted using RevMan (Version 5.4, The Cochrane Collaboration, 2020). The participants were grouped into dental prosthesis users and non-users. The association between dental prosthesis use and each outcome was separately examined. Hypertension and physical function outcomes were measured as binary variables and summarized as odds ratios (ORs) with 95% confidence intervals (CIs). When studies did not report direct comparisons between denture users and non-users or reported time-to-event analyses, unadjusted ORs were calculated from the raw frequency or cumulative incidence data presented in the original articles. Pooled estimates were generated using a random effects model to account for between-study heterogeneity. Statistical heterogeneity was assessed using the I² statistic, which quantifies the proportion of variation due to true differences, rather than chance. Statistical significance was set at *P* < .05. Given the variability in exposure definitions and analytical adjustments across studies, this scoping review included an exploratory, hypothesis-generating quantitative synthesis to summarize the direction and patterns of association and was not intended as a confirmatory meta-analysis.

## Results

### Overview of included populations and CVD definitions

**Fig. 1:**
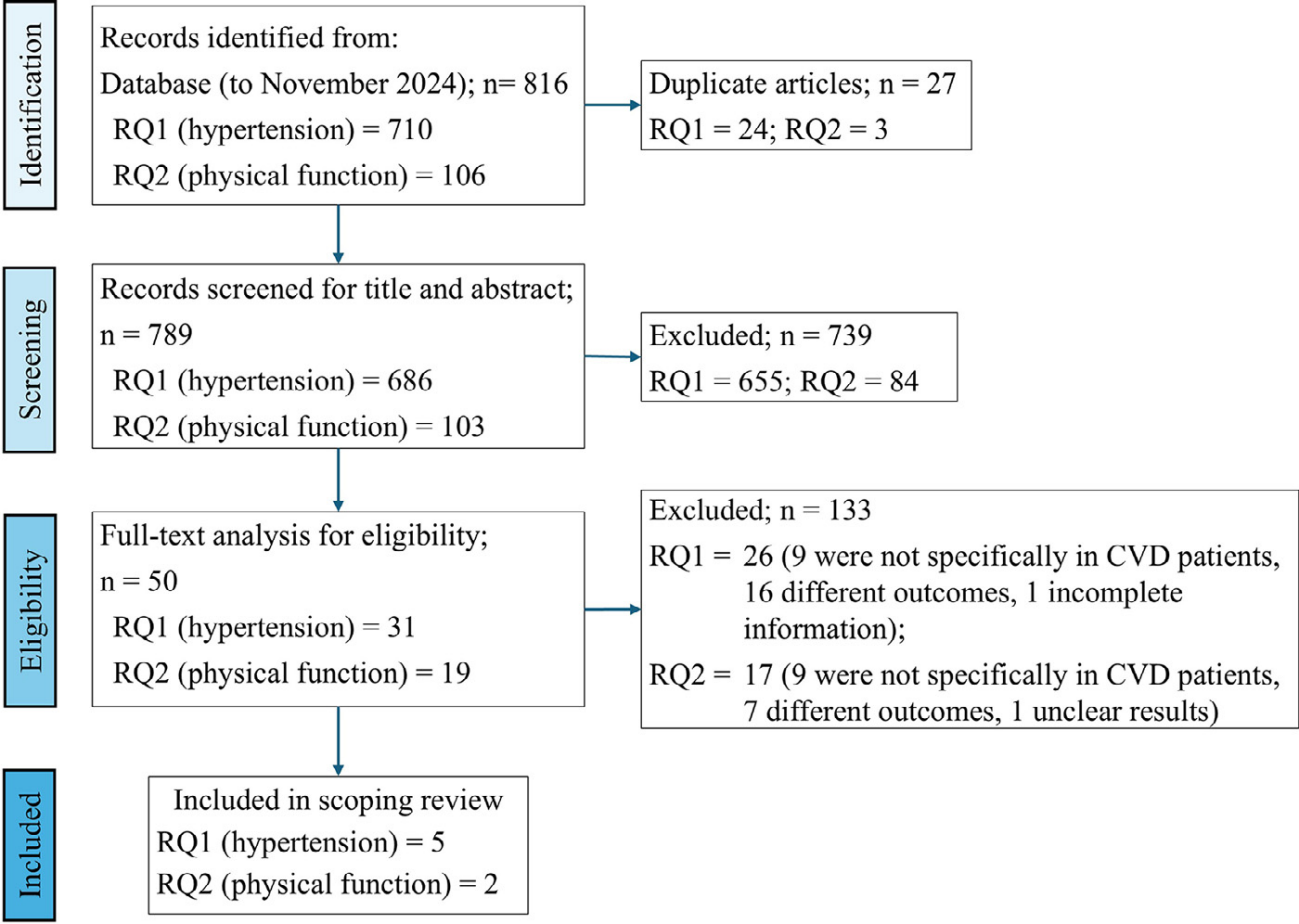
PRISMA flow diagram depicting the study selection process.

The literature screening and selection process are summarized in Figure 1. The database search initially yielded 816 publications. After screening titles and abstracts, 50 studies were selected for full-text review. Of these, 7 studies met the eligibility criteria and were included in this review (Table 3).

**Table 3:** Summary of risk of bias and significance and strength of evidence.

RQ	First author -Year -Country	Study design -Follow-up period	Exposure Group/Comparator (n)	Prosthesis Type -Assessment method	Population category (CVD definition)	Main Outcome -Assessment method	Key Findings	Adjusted for confounders: Yes/No
RQ1	Abe (2022)^a^ Japan	Cross-sectional study	0-19 teeth with dentures (197)/without dentures (10) 20-27 teeth with dentures (101)/without dentures (100)	Not specified Examined by a dental hygienist	Hypertensive older adults/antihypertensive medication users	Hypertension Interviewed and BP measured by trained nurses	Denture use appeared to attenuate the association between fewer remaining teeth and hypertension prevalence.	No
	Carra (2021)^b^ France	Cohort study 7 years	Teeth replaced with dental prostheses (21,684)/Teeth not replaced with dental prostheses (10,464)	Not specified Self-reported	Arterial hypertensive older adults/antihypertensive medication users	Arterial hypertension Self-reported	Unreplaced tooth loss was associated with a higher incidence of arterial hypertension during follow-up.	No
	Dai (2023) China	Cohort study (baseline)	Denture use (1,896)/No denture use (3,507)	Not specified Self-reported	General older population (CVD not specified)	Hypertension Self-reported	Hypertension prevalence was higher among denture users than among non-users.	No
	Liu (2024) China	Cross-sectional study	Fit denture (157,371)/Ill-fit denture (1,288)	Fixed and Removable Denture (Fit and Ill-fit) Examined by dentists	General older population (CVD not specified)	Hypertension BP measured	Ill-fitting prostheses were associated with a higher risk of hypertension and higher systolic and diastolic blood pressure levels.	No
	Watt (2012)^c^ Scotland	Cohort study (baseline)	Natural teeth with denture (3,615)/Edentate (3,240)	Not specified Self-reported	General older population (CVD not specified)	Hypertension Doctor’s diagnosis or BP measured in clinic	Hypertension prevalence was higher among edentulous participants than among those with natural teeth and denture use.	No
RQ2	Kimble (2023)^d^ United Kingdom	Cross-sectional study	Use PD only (245)/Not use with <21teeth (141) Use CD only (143)/Not use with <21teeth (141) Use PD and CD (53)/Not use with <21teeth (141)	Removable denture (Partial denture only; Full denture only; Partial denture and Complete denture) Examined and questionnaire	General population of older men from BRHS study (CVD not specifically defined)	Physical frailty Fried Frailty Phenotype (FFP) questionnaire	Participants with impaired natural dentition and no denture use showed the highest frailty prevalence, whereas complete denture wearers showed a lower frailty prevalence.	No
	Ogawa (2021)^e^ Japan	Cohort study 4 years	Partially or fully edentulous with dentures (158)/functionally inadequate with no denture (299)	Not specified Examined by a trained dentist and dental hygienist	Hospitalized patients with clinically diagnosed cardiovascular disease	Physical frailty Short Physical Performance Battery (SPPB) questionnaire	Inadequate occlusion without dentures was associated with a higher risk of frailty during follow-up.	No

Abbreviations: CVD, cardiovascular disease; PD, partial denture; CD, complete denture; ND, natural dentition; BRHS, British regional heart study

a Participants were categorized into 5 groups; those with ≥28 teeth were excluded from this study.

b Participants were categorized into 3 groups based on responses to a question about having missing teeth replaced by dental prostheses; responses of “I don’t know” were excluded.

c Participants were categorized into 3 groups; individuals with only natural teeth were excluded.

d Participants were categorized into 5 groups based on denture type; individuals with functional dentition (≥21 teeth) and no denture use were excluded.

e Participants were categorized into 5 groups based on occlusal support; individuals with natural dentition providing adequate function were excluded. Prosthesis type refers to the primary dental prosthesis intervention assessed as removable, fixed, or implant-supported dentures. Key findings summarize the main reported associations. Adjustment for confounders indicates whether the effect estimate used in this review was derived from an analysis that controlled for variables that might influence the outcomes. Estimates reconstructed from raw data were classified as unadjusted.

The included publications, dated between 2012 and 2023, were observational. Four were cohort studies; however, 2 assessed hypertension using only baseline data and were therefore treated as cross-sectional analyses in this review. The remaining 3 studies were cross-sectional. No randomized controlled trials or other interventional studies were identified, underscoring the limited evidence base and methodological constraints in this research area.

All included studies focused on populations with CVD or at elevated cardiovascular risk; however, the definitions of CVD and cardiovascular risk status varied substantially across studies. The included populations could be broadly classified into 3 categories: (1) hospital-based patients with clinically diagnosed cardiovascular disease, (2) community-dwelling older adults in whom cardiovascular risk was defined primarily by hypertension or antihypertensive medication use, and (3) general population cohorts in which CVD status was not explicitly defined. This clinical heterogeneity in cardiovascular status likely contributed to the variability in effect estimates across studies.

Hypertension and physical function (including incidentally reported frailty) were the primary outcomes assessed, consistent with predefined research questions.

### Dental prostheses and hypertension (RQ1)

Five studies investigated the association between dental prosthesis use and hypertension, including 2 cross-sectional and 3 cohort studies. Among the cohort studies, two relied solely on baseline data, whereas one longitudinal study^26^ prospectively examined the incidence of hypertension.

Definitions of dental prosthesis exposure varied across studies, and the type of dental prosthesis was frequently not specified. Abe et al.^19^ examined denture use in relation to the number of remaining teeth, whereas Carra et al.^26^ and Watt et al.^27^ classified denture use based on whether dental prostheses replaced missing teeth. Only one study assessed the dental prosthesis condition, while Liu et al.^28^ differentiated between well-fitting and ill-fitting prostheses.

The outcomes of hypertension were variably defined. Some studies assessed hypertension using measured blood pressure or a doctor’s diagnosis, whereas others relied on self-reported hypertension. Some studies^19^^,^^27^ reported a lower hypertension prevalence among individuals using dental prostheses compared with non-users, whereas Dai et al.^29^ reported a higher prevalence of hypertension among denture users.

The effect estimates included in the pooled analysis were derived from unadjusted data. Although some original studies reported adjusted models, odds ratios were reconstructed from raw data to ensure consistency across studies. Adjusted estimates were not pooled because covariate selection and model specifications differed substantially across studies, limiting comparability. Therefore, unadjusted effect estimates were used to provide a consistent exploratory synthesis, and all the estimates were classified as unadjusted.

**Fig. 2:**
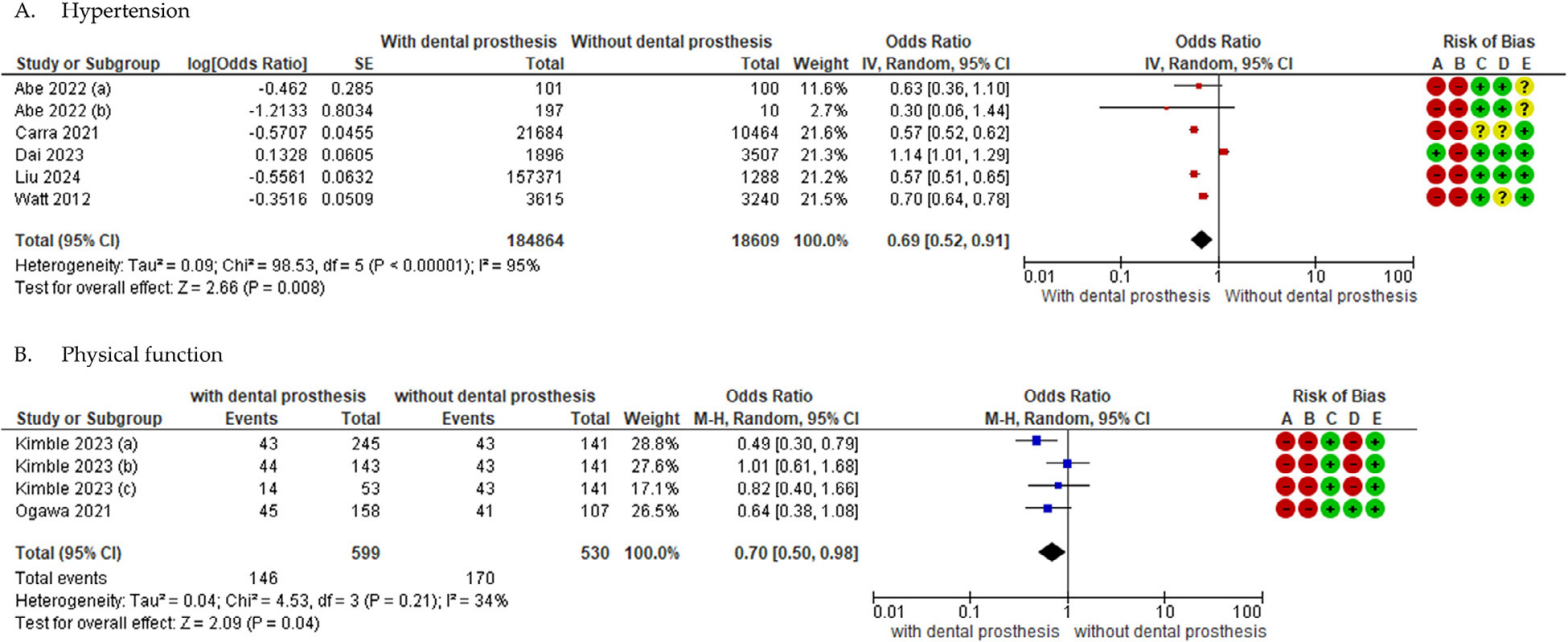
Forest plot of the association between dental prosthesis use, hypertension, and physical function. (A) Hypertension and (B) Physical function: unadjusted odds ratios (ORs) with 95% confidence intervals (CIs) for each outcome. Each plot presents the individual study estimates, unadjusted pooled effect sizes, and heterogeneity statistics (I²). Risk of bias was indicated using color-coded markers: green (low risk), yellow (unclear risk), and red (high risk). Pooled estimates are based on unadjusted data and should be interpreted as exploratory. Notes: Abe 2022: (A) denture use with 0-19 remaining teeth; (B) denture use with 20-27 remaining teeth. Kimble 2023: (A) partial denture only; (B) complete denture only; (C) mixed partial and complete dentures.

**Fig. 3:**
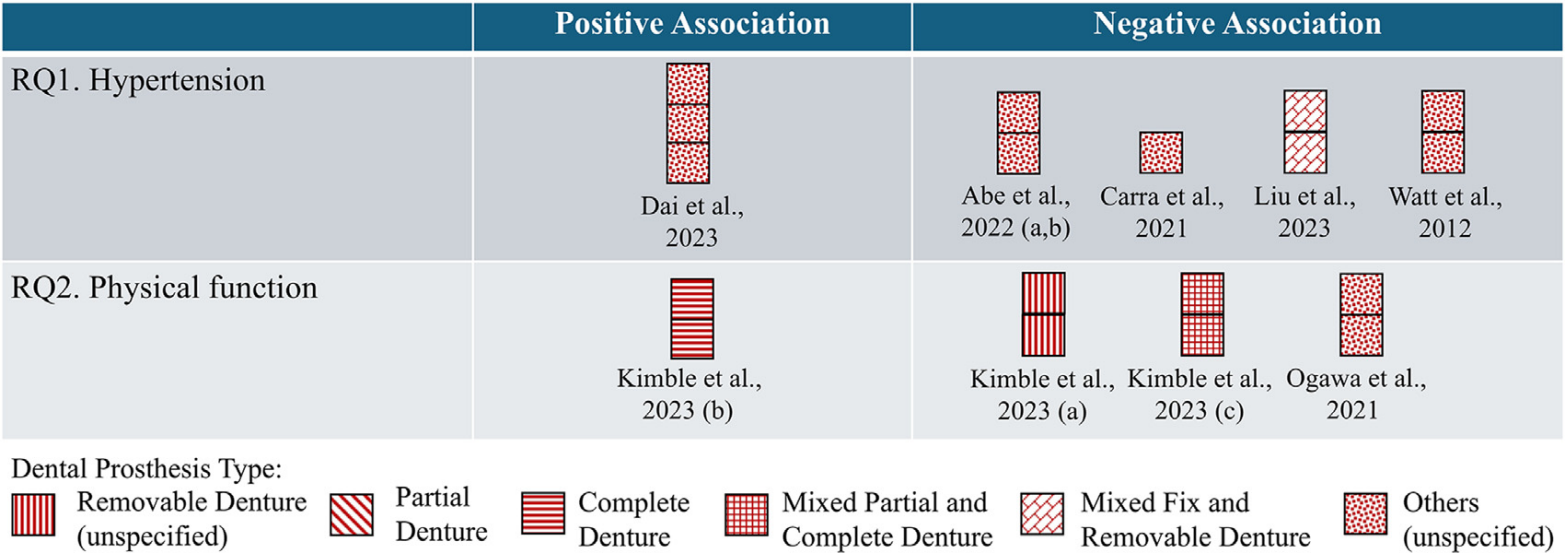
Summary of evidence for associations between dental prosthesis use, hypertension, and physical function. Frailty findings should be interpreted as secondary and exploratory outcomes within the physical function domain. Bar height indicates the risk of bias: 3 blocks, low risk; 2 blocks, serious risk; 1 block, very serious risk. Positive Association: Dental prosthesis use is associated with increased risk or occurrence of hypertension or frailty. Negative Association: Dental prosthesis use is associated with a decreased risk or occurrence of hypertension or frailty (potential protective effect).

The pooled analysis (Figures 2A and 3) included 6 comparisons from 5 studies, with one study contributing to two independent comparisons. Dental prosthesis use was associated with a lower odds of hypertension, based on unadjusted pooled odds ratio (OR = 0.69; 95% CI: 0.52-0.91; Z = 2.66; *P* = .008). The substantial heterogeneity observed (I² = 95%). Sensitivity or subgroup analyses were not feasible due to the small number of included studies and the lack of consistent reporting of prosthesis characteristics and adjustment for confounders. Consequently, the pooled estimates should be interpreted as exploratory and hypothesis-generating, rather than confirmatory.

### Dental prostheses and physical function in patients with CVD (RQ2)

Two studies examined the association between dental prostheses and physical function in patients with CVD. Although frailty was not included as a search term, it emerged as an outcome in both studies and was therefore treated as a secondary, exploratory component of the physical domain.^30^^,^^31^

Dental prosthesis exposure was defined differently across studies. Kimble et al.^30^ differentiated between the dental prosthesis type and partial and complete dentures. However, Ogawa et al.^31^ did not specify the type of denture. None of the studies assessed the condition of the dental prosthesis.

The frailty outcomes were assessed differently in the studies. Based on the Fried Frailty Phenotype (FFP), Kimble et al.^30^ observed that frailty prevalence was highest among individuals with impaired natural dentition and no dental prostheses, whereas complete denture wearers showed frailty comparable to those with impaired natural dentition. In contrast, based on the Short Physical Performance Battery (SPPB), Ogawa et al.^31^ reported lower odds of frailty among dental prosthesis users during follow-up.

The effect estimates included in the pooled analysis were derived from unadjusted data. Although adjusted analyses were reported in the original studies, odds ratios were reconstructed from raw data to allow a consistent comparison between denture users and non-users.

The pooled analysis of these two studies (Figure 2B and 3) showed lower odds of frailty among dental prosthesis users, based on unadjusted pooled odds ratio (OR = 0.70; 95% CI: 0.50-0.98; Z = 2.09; *P* = .04), with low heterogeneity (Tau² = 0.04; Chi² = 4.53, df = 3; *P* = .21; I² = 34%). Sensitivity analyses were not feasible because of the small number of included studies and variability in exposure definitions, outcome measures, and confounder adjustments. Therefore, pooled estimates should be interpreted as exploratory and hypothesis-generating.

### Evaluation of bias risk and quality of evidence

Figure 3 and Table 4 summarize the risk of bias, strength, and certainty of the evidence for each research question.

**Table 4:** Summary of risk of bias and significance and strength of evidence.

Outcome (Study design/number of studies)	OR/HR/MD (95%CI)	Risk of Bias	Inconsistency	Indirectness	Imprecision	Publication Bias	Certainty of the evidence (GRADE)
Hypertension (Cross-sectional/2; Cohort/3)	0.69 (0.52 to 0.91)	-1	-2	0	-1	0	Very low^a^^,^^b^
Physical function (Cross-sectional/1; Cohort/1)	0.70 (0.50 to 0.98)	-1	-1	0	0	0	Low^a^

a Serious risk of bias due to selection and performance bias.

b Serious unexplained inconsistency (high heterogeneity I^2^ = 95%, *P* value < .00001). Scoring scale: 0 = low risk; -1 = unclear; -2 = high risk. Certainty of evidence (GRADE): Very low = one or more domains with very serious concerns; Low = serious but not very serious concerns.

For RQ1 (hypertension), considerable variability was observed in sample size, study design, and definitions of dental prosthesis use. None of the studies adequately adjusted for potential confounders such as age, smoking status, or comorbidities, thereby introducing a risk of residual bias. Selection bias was also possible, as most studies were based on outpatient populations, and measurement bias may have arisen from reliance on self-reported blood pressure or prosthesis status. The high heterogeneity observed in the pooled analysis (I² = 95%) further reduced the confidence. Overall, the evidence was rated very low in certainty.

For RQ2 (physical function), it is important to note that frailty outcomes were incidentally identified rather than systematically searched, thereby limiting the comprehensiveness of the evidence in this domain. Furthermore, the two included studies used different frailty assessment tools (FFP vs. SPPB), which impedes direct comparison. Both studies failed to adequately control for key confounders, including age, comorbidities, medication use, and nutritional status. Additionally, the definitions of dental prosthesis type and condition were inconsistent. Although both studies suggested a protective association, the evidence was rated with low certainty due to methodological limitations and a restricted evidence base.

## Discussion

In the present review, hypertension was intentionally treated as a clinical outcome, rather than a cardiovascular risk factor. This conceptualization is particularly relevant in older adults and in patients with established CVD, in whom blood pressure is routinely monitored and therapeutically modified as part of disease management. This approach parallels the treatment of frailty in this review, which was considered a functional outcome reflecting the disease burden and progression in patients with CVD. Although this review provides a structured overview of existing evidence, the pooled estimates were derived from unadjusted data and may be influenced by residual confounding and population heterogeneity; therefore, they should be interpreted as exploratory and hypothesis-generating, rather than confirmatory.

### Dental prostheses and hypertension

Several studies have suggested that dental prosthesis use may be associated with lower odds of hypertension, although the findings are inconsistent across studies. Collectively, the evidence points toward a potential role of functional oral rehabilitation, rather than its presence alone, in supporting blood pressure control among older adults and patients with CVD.

From a mechanistic perspective, well-fitted dental prostheses may restore masticatory function, improve dietary intake, and reduce systemic inflammation, thereby contributing to improved blood pressure regulation. In contrast, ill-fitting dentures may promote plaque accumulation and microbial dysbiosis, which trigger systemic inflammation and pathways that may be implicated in hypertension development^26^^,^^28^ Carra et al.^26^ provided the strongest temporal evidence by demonstrating an increased risk of hypertension in individuals with unreplaced tooth loss. Other studies have indicated that preserving masticatory performance with dental prostheses also helps prevent hypertension, even in older adults with substantial tooth loss.^19^

Several methodological factors limit the interpretation of these findings. Across studies, dental prosthesis exposure was heterogeneously defined, with limited specification of dental prosthesis type and inconsistent definition of the comparator group, ranging from edentulous individuals to those with unreplaced tooth loss. Relevant characteristics such as dental prosthesis fit, duration of use, and hygiene practices were rarely reported, limiting accurate exposure classification. This is particularly relevant given that edentulism, dental prosthesis use, and masticatory function represent distinct conditions with potentially different implications for cardiovascular outcomes. Hypertension outcomes were assessed in various ways, ranging from measured blood pressure to self-reported diagnoses. Furthermore, pooled effect estimates were derived from unadjusted data, and residual confounding cannot be ruled out. The observed association between dental prosthesis use and lower odds of hypertension may reflect confounding by socioeconomic status, access to care, or health-seeking behavior. These findings, therefore, warrant cautious interpretation as exploratory and hypothesis-generating rather than confirmatory.

### Dental prostheses and physical function in patients with CVD

Dental prostheses may also help preserve physical function and mitigate functional decline in patients with CVDs. Although frailty was not a predefined search outcome, it emerged in two of the included studies,^30^^,^^31^ highlighting the potential relevance of oral rehabilitation to functional health in this population.

Across studies, a consistent pattern was observed, whereby edentulous individuals without prostheses exhibited greater frailty or functional impairment, while denture wearers demonstrated functional capacity comparable to that of individuals with natural dentition. In a cross-sectional study by Kimble et al.,^30^ complete denture use was associated with lower frailty prevalence, whereas in a longitudinal study by Ogawa et al.,^31^ dental prosthesis use appeared to protect against frailty progression over time. These findings imply that prosthodontic rehabilitation may help maintain physical independence through mechanisms involving masticatory efficiency, dietary adequacy, and psychosocial engagement.

However, the interpretation of this evidence was constrained by methodological limitations. Only two studies were identified; frailty was assessed using different instruments (FFP vs. SPPB), and dental prosthesis type and condition were insufficiently reported. Notably, the comparator groups varied across studies, ranging from individuals with reduced natural dentition without dental prosthesis to those with functionally inadequate occlusion, making it difficult to isolate the independent effect of dental prosthesis use on physical function outcomes. This reflects a broader conceptual challenge, as dental prosthesis use alone may not adequately capture functional oral status, given that oral function is influenced by multiple factors beyond dental prosthesis presence. Furthermore, the adjustment for confounding factors was limited, and the study designs differed substantially. As a result, although both studies point toward a beneficial association, the evidence base remains preliminary and should be regarded as exploratory and hypothesis-generating rather than confirmatory, emphasizing the need for more rigorous longitudinal studies.

### Limitations and future directions

This study had several limitations. First, the term ‘CVD or at risk for CVD’ encompasses heterogeneous populations ranging from diagnosed CVD patients to community-dwelling older adults with hypertension, which may have introduced clinical heterogeneity. Second, the evidence base was limited by a small number of included studies and a search strategy restricted to two databases, which may have resulted in incomplete identification of relevant studies and potential selection bias. Third, substantial heterogeneity was observed across studies, likely reflecting differences in study design, population characteristics, inconsistent reporting of dental prosthesis features (type, fit, and duration of use), and inconsistent use of frailty assessment instruments, which limited comparability. Moreover, edentulism, dental prosthesis use, and masticatory function represent distinct clinical conditions that were not always clearly differentiated across studies, potentially reducing the interpretability of the observed associations. Fourth, pooled estimates were derived from unadjusted data, increasing the risk of residual confounding and making it difficult to determine the independent contribution of dental prosthesis use. The observed associations may reflect differences in socioeconomic status, access to care, or health-seeking behaviour rather than a true protective effect. As most studies were conducted in East Asian populations, these findings may not be generalizable to other regions. Overall, results should be interpreted with caution, regarded as exploratory and hypothesis-generating rather than confirmatory.

Based on the findings and limitations of this study, several priorities have emerged for future research. Well-designed prospective studies are required to clarify the temporal relationship between prosthodontic rehabilitation and cardiovascular and functional outcomes. Standardized definitions of dental prostheses exposure, including type, fit, duration of use, and quality, as well as consistent outcome measurement, would enhance cross-study comparability. Future investigations should also incorporate comprehensive adjustment for confounders, particularly socioeconomic status, nutritional intake, comorbidities, other oral health factors, psychosocial factors, and inflammatory markers, to explore potential mediating pathways. Research should also include diverse populations beyond East Asian settings and examine a broader range of dental prostheses to better reflect current clinical practice and improve generalizability.

### Clinical implications

Dental prosthetic treatment may support systemic and functional health in adults with or at risk of cardiovascular disease by restoring mastication and facilitating adequate nutrition. Consideration of the function and fit of dental prostheses may be important when managing patients with cardiovascular risk. The integration of prosthodontic care into multidisciplinary cardiovascular and geriatric management may help support functional independence and healthy aging.

## Conclusions

Current evidence suggests that dental prostheses may provide benefits beyond oral rehabilitation, with a potential association with lower hypertension risk and preservation of physical function in adults with or at risk of cardiovascular disease. These findings support the consideration of oral rehabilitation as a part of comprehensive care strategies for aging populations.

However, these findings should be interpreted as exploratory and hypothesis-generating rather than confirmatory, given methodological heterogeneity, small sample sizes, and predominantly observational study designs with insufficient control for confounding variables. Further well-designed longitudinal and interventional studies are required to clarify the causal pathways and to optimize the integration of prosthodontic care into clinical practice.

## Funding

The authors declare no financial support for the research, authorship, or publication of this article.

## Author contribution

**Monika Werdiningsih**: Conceptualization, Methodology, Data curation, Formal analysis, Visualization, Writing – original draft preparation. **En-Chih Weng**: Conceptualization, Methodology, Formal analysis, Investigation, Writing – review and editing**. Ma. Therese Sta. Maria**: Formal analysis, Investigation. **Yoko Hasegawa**: Conceptualization, Methodology, Supervision, Writing – review and editing. **Kazuhiro Hori**: Supervision, Writing – review and editing. **Yasuyuki Nagasawa**: Clinical expertise, Interpretation of data, Writing – review and editing. **Ken Shinmura**: Clinical expertise, Interpretation of data, Writing – review and editing. All authors read and approved the final version of the manuscript.

## Conflict of interest

None disclosed.
